# Supplementary material for: Saccharomyces boulardii (CNCM I-745) improves intestinal damage in sepsis by remodeling intestinal flora structure
Source: Front Cell Infect Microbiol. 2025 Jul 31;15:1602792. doi: 10.3389/fcimb.2025.1602792 (PMC12350329; doi:10.3389/fcimb.2025.1602792)
Supplement: Supplementary file 1 [file DataSheet1.zip › summary/summary/8_Advanced_analyse/2_PICRUSt2_result/KEGG/Group.DW4_vs_DW0_vs_CW4_vs_CW0_vs_BW4_vs_BW0_vs_AW4_vs_AW0/PICRUSt2_KEGG_level2_Group.DW4_vs_DW0_vs_CW4_vs_CW0_vs_BW4_vs_BW0_vs_AW4_vs_AW0_diff.pdf]

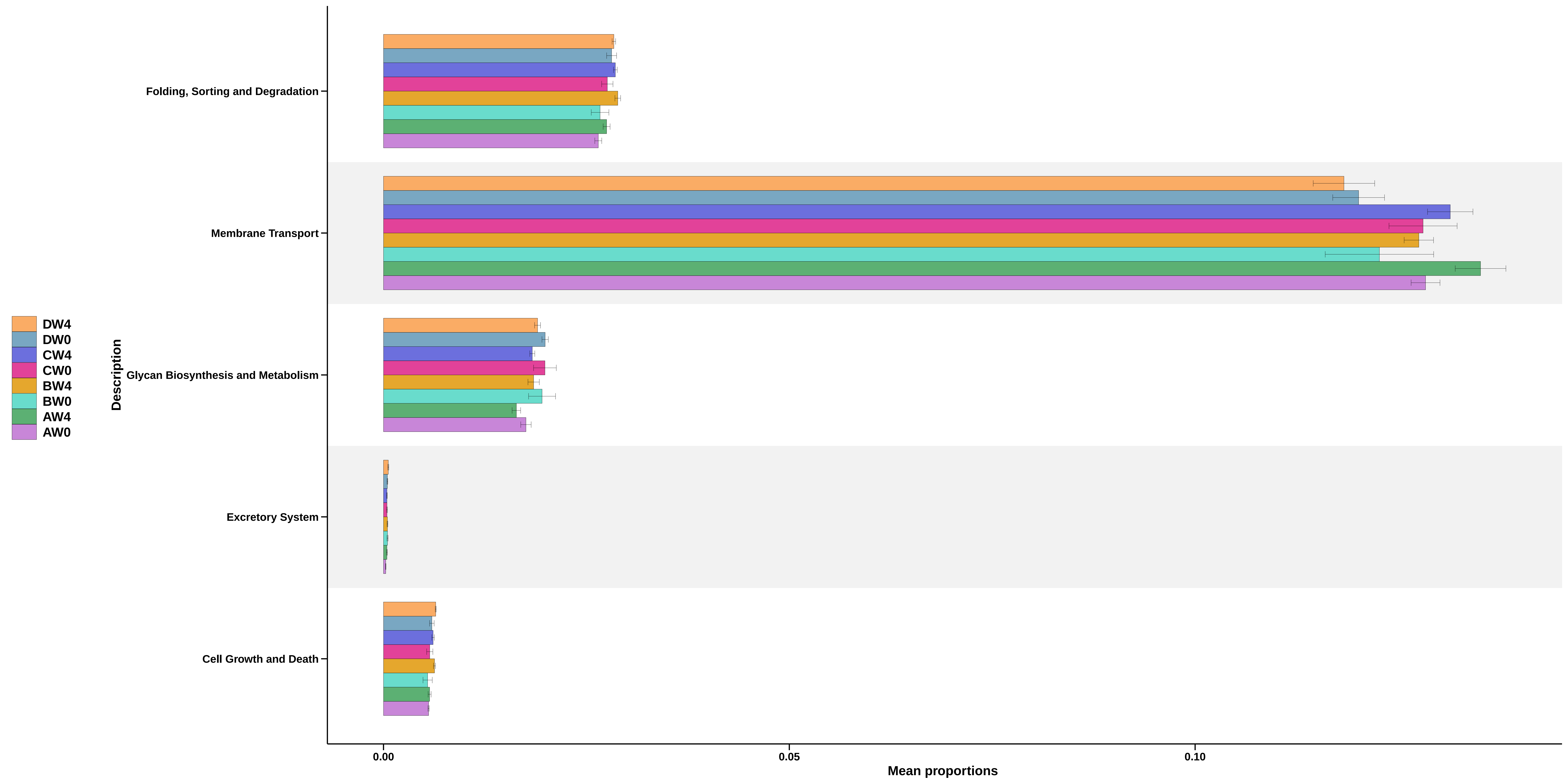

DW4
DW0
CW4
CW0
BW4
BW0
AW4
AW0
Description
Folding, Sorting and Degradation
Membrane Transport
Glycan Biosynthesis and Metabolism
Excretory System
Cell Growth and Death
0.00
0.05
0.10
Mean proportions
